# Red Wine Polyphenols Prevent Metabolic and Cardiovascular Alterations Associated with Obesity in Zucker Fatty Rats (Fa/Fa)

**DOI:** 10.1371/journal.pone.0005557

**Published:** 2009-05-18

**Authors:** Abdelali Agouni, Anne-Hélène Lagrue-Lak-Hal, Hadj Ahmed Mostefai, Angela Tesse, Paul Mulder, Philippe Rouet, Franck Desmoulin, Christophe Heymes, Maria Carmen Martínez, Ramaroson Andriantsitohaina

**Affiliations:** 1 INSERM, U771, Angers, France; 2 CNRS UMR, 6214, Angers, France; 3 Université d'Angers, Angers, France; 4 INSERM, U644, Rouen, France; 5 INSERM, U858, Toulouse, France; 6 Université Paul Sabatier, Institut Fédératif de Recherche 31, Toulouse, France; 7 INSERM, U689, Paris, France; University of Camerino, Italy

## Abstract

**Background:**

Obesity is associated with increased risks for development of cardiovascular diseases. Epidemiological studies report an inverse association between dietary flavonoid consumption and mortality from cardiovascular diseases. We studied the potential beneficial effects of dietary supplementation of red wine polyphenol extract, Provinols™, on obesity-associated alterations with respect to metabolic disturbances and cardiovascular functions in Zucker fatty (ZF) rats.

**Methodology/Principal Findings:**

ZF rats or their lean littermates received normal diet or supplemented with Provinols™ for 8 weeks. Provinols™ improved glucose metabolism by reducing plasma glucose and fructosamine in ZF rats. Moreover, it reduced circulating triglycerides and total cholesterol as well as LDL-cholesterol in ZF rats. Echocardiography measurements demonstrated that Provinols™ improved cardiac performance as evidenced by an increase in left ventricular fractional shortening and cardiac output associated with decreased peripheral arterial resistances in ZF rats. Regarding vascular function, Provinols™ corrected endothelial dysfunction in aortas from ZF rats by improving endothelium-dependent relaxation in response to acetylcholine (Ach). Provinols™ enhanced NO bioavailability resulting from increased nitric oxide (NO) production through enhanced endothelial NO-synthase (eNOS) activity and reduced superoxide anion release via decreased expression of NADPH oxidase membrane sub-unit, Nox-1. In small mesenteric arteries, although Provinols™ did not affect the endothelium-dependent response to Ach; it enhanced the endothelial-derived hyperpolarizing factor component of the response.

**Conclusions/Significance:**

Use of red wine polyphenols may be a potential mechanism for prevention of cardiovascular and metabolic alterations associated with obesity.

## Introduction

Obesity is a fast growing problem that is reaching epidemic proportions worldwide, and is associated with an increased risk of premature death [1]. Individuals with a central deposition of adipose tissue display elevated cardiovascular morbidity and mortality, including stroke, congestive heart failure, myocardial infarction and cardiovascular death, and this is independent of the association between obesity and other cardiovascular risk factors [2]. Obesity, in particular abdominal obesity, was pointed out as a primary contributor to acquired insulin resistance, as increasing adiposity is correlated with impaired insulin action [3]. Endothelial dysfunction, an independent predictor of cardiovascular events [4], has been consistently associated with obesity and the metabolic syndrome [5] in a complex interplay with insulin resistance [6]. Deficiency of endothelial nitric oxide (NO) is believed to be the primary defect that links insulin resistance and endothelial dysfunction [7].

Epidemiological studies report an inverse association between dietary polyphenol consumption and mortality from cardiovascular diseases [8]. In the past decade, red wine polyphenolic compounds, including Provinols™, have been shown to exert numerous biological effects that might participate in cardio- and vasculo-protection. Polyphenols possess anti-aggregatory platelet activity, antioxidant and free radical scavenging properties [9], [10]. Furthermore, red wine and its polyphenolic constituents possess lipid- and lipoprotein-lowering effects [9]. Moreover, Provinols™ is a powerful vasodilator through the generation of NO and can act on the expression of genes protective of the cardiovascular system [11]. Due to these pleiotropic properties, polyphenols may be good candidates to correct metabolic and cardiovascular alterations associated with obesity and metabolic syndrome.

In the present work, we studied the potential beneficial effects of dietary supplementation of the red wine polyphenol extract, Provinols™, in an experimental model of obesity and metabolic syndrome with an emphasis on glucose and lipid metabolisms, cardiac and endothelial functions. Of particular interest is to study the cellular pathways related to nitrosative and oxidative stresses in blood vessels. For this purpose, we used the Zucker fatty (ZF) rat, a widely used animal model of obesity and type 2 diabetes, which presents many of the human metabolic syndrome features such as insulin resistance, dyslipidemia, hyperinsulinemia [12], [13] and, in some colonies, hypertension that develops by 4–5 months of age [14]. On the other hand, their lean littermates are insulin-sensitive, normoinsulinemic, normotensive, and have normal lipid profiles and glucose tolerance. Furthermore, this model presents a loss of functional mutation in the leptin receptor [15], [16].

## Methods

### Animals and experimental protocol

The university of Angers ethical committee approved the present protocol. All animal studies were carried out using approved institutional protocols and were conformed the *Guide for the Care and Use of Laboratory Animals* published by US National Institutes of Health (NIH Publication No. 85-23, revised 1996). Twenty-four ZF rats and twelve of their lean littermate non-obese rats (Charles River; L'Arbresle, France) received for eight weeks either a control diet or a diet containing Provinols™ (Société Française de Distilleries; Vallon Pont d'Arc, France) at the dose of 20 mg/kg/day. The dose of Provinols™ was described to induce cardiovascular effects, including improvement of endothelial function [17] and prevention of the increase in blood pressure in NO-deficient hypertensive rats [18] and protection of both cardiac ischemia [19] and stroke [20]. Eight weeks of treatment has been reported to protect against deleterious effects of hypertension [21] and chronic-stress exposure [22].

### Measurement of blood pressure

Blood pressure was measured using the tail-cuff technique weekly. Each rat was trained to the tail-cuff technique for 2 days before each measurement recorded with a Physiograph Desk Model and an Electro-Sphygmomanometer (BIOSEB; Paris, France). Five separate measurements were made on conscious rats for systolic blood pressure and heart rate determinations.

### Echocardiographic examination

Transthoracic echocardiography was performed using an ATL-HDI 5500 ultrasound machine equipped with a 8-MHz imaging transducer in rats anesthetized with pentobarbital sodium (50 mg/kg *ip*). Briefly, a two-dimensional short axis view of the left ventricle was obtained at the level of the papillary muscle in order to record M-mode tracings. Diastolic left ventricular dimension (LVDd) and systolic left ventricular dimension (LVDs) were measured by the American Society of Echocardiology leading-edge method from at least 3 consecutive cardiac cycles. Doppler cardiac output was calculated by the following formula: Cardiac output = Л×D^2^/4×IVTA_o_, where D represents the diameter of the aortic left ventricular outflow tract and IVTA_o_ the velocity-time integral in the left ventricular outflow tract. Also, total arterial peripheral resistances were calculated from blood pressure and cardiac output.

### Circulating parameters measurement

Rats were anesthetized with isoflurane and blood was harvested from abdominal aorta. The plasma was separated for circulating glucose, fructosamine, total cholesterol, low density lipoprotein (LDL)-cholesterol, high density lipoprotein (HDL)-cholesterol, triglycerides, creatinin, and uric acid analysis (technical platform of Toulouse Midi-Pyrénées Genopole® IFR31; Toulouse, France).

### Vascular reactivity

Aortic and small mesenteric artery (SMA) rings (1.5–2 mm long) were obtained from ZF and lean rats having received Provinols™ or a vehicle. Then, they were mounted on a wire myograph (Danish MyoTechnology, Aarhus, Denmark) filled with physiological salt solution (PSS) as previously described [23]. Endothelium-dependent vasodilatation in response to acetylcholine (Ach, 1 nM to 10 µM) was studied in aortas and SMA with functional endothelium pre-contracted with the thromboxane A_2_ agonist (9, 11-dideoxy-11a, 9a-epoxymethanoprostaglandin F2-α) U46619 (Sigma-Aldrich; St. Quentin, Fallavier, France) at 80% of their maximal response. In SMA from ZF rats the different components of the vaso-relaxation were determined as follows: the NO-dependent dilation was measured in the presence of a non selective cyclo-oxygenase (COX) inhibitor, indomethacin, (INDO, 100 µM; Sigma-Aldrich), apamin (100 nM; Sigma-Aldrich) and charybdotoxin (50 nM; Sigma-Aldrich). The endothelium-derived hyperpolarizing factor (EDHF) component was assessed in the presence of the NO synthase inhibitor N^ω^-Nitro-L-arginine methyl ester (L-NAME, 100 µM; Sigma-Aldrich) and INDO (100 µM).

### Nitric oxide (NO) and superoxide anion (O_2_^−^) determination by electron paramagnetic resonance (EPR)

Aortas, carotid arteries and SMA were dissected and incubated for NO production for 30 min in Krebs–Hepes buffer containing: BSA (20.5 g/L), CaCl_2_ (3 mM) and L-Arginine (0.8 mM) (Sigma-Aldrich). NaDETC (1.5 mM) and FeSO_4_.7H_2_O (1.5 mM) (Sigma-Aldrich) were separately dissolved under argon gas bubbling in 10 mL volumes of ice-cold Krebs–Hepes buffer. These were rapidly mixed to obtain a pale yellow-brown opalescent colloid Fe(DETC)_2_ solution (0.4 mM), which was used immediately. The colloid Fe(DETC)_2_ solution was added to vessels and incubated for 45 minutes at 37°C. Then, arteries were immediately frozen in plastic tubes using liquid N_2_. NO measurement was performed on a table-top x-band spectrometer Miniscope (Magnettech, MS200; Berlin, Germany). Recordings were made at 77°K, using a Dewar flask. Instrument settings were 10 mW of microwave power, 1 mT of amplitude modulation, 100 kHz of modulation frequency, 150 s of sweep time and 3 scans. Signals were quantified by measuring the total amplitude, after correction of baseline as done previously [23]. Values are expressed in unit/mg weight of dried tissue.

For O_2_^−^ spin-trapping, aortas, carotid arteries and SMA were dissected and allowed to equilibrate in deferoxamine-chelated Krebs-Hepes solution containing 1-hydroxy-3methoxycarbonyl-2,2,5,5-tetramethylpyrrolidin (CMH, Noxygen; Denzlingen, Germany) (500 µM), deferoxamin (25 µM, Sigma-Aldrich) and DETC (5 µM, Sigma-Aldrich) under constant temperature (37°C) for 60 minutes. Then, they were frozen in liquid N_2_ and analyzed in a Dewar flask by EPR. Values are expressed in unit/mg weight of dried tissue.

### Western Blotting

Aorta was dissected, homogenized and lysed. Proteins (80 µg) were separated on 10% SDS-PAGE. Blots were probed with anti-endothelial NOS (eNOS), anti-phospho-eNOS Ser 1177, anti-phospho-eNOS Thr 495 (Cell Signaling; Beverly, MA), anti-caveolin-1 (BD Biosciences; San Jose, CA), Mn-superoxide dismutase (SOD), Cu/Zn-SOD, extracellular (Ec)-SOD (Stressgen Biotechnologies Corporation; Victoria, Canada), anti-gp91^phox^ (Nox-2; BD Biosciences), anti-Nox-1 and anti-Nox-4 antibodies.

### Data analysis

Data are expressed as mean±SEM, and *n* represents the number of experiences. Statistical analyses were performed using a one way analysis of variance (ANOVA), and Mann-Whitney U tests or ANOVA for repeated measures and subsequent Bonferroni post hoc test. *P*<0.05 was considered to be statistically significant.

## Results

### Provinols™ improve circulating parameters in ZF rats

ZF rats weighed significantly more than their lean controls and Provinols™ treatment did not affect body weight of either group (data not shown).

**Figure 1:**
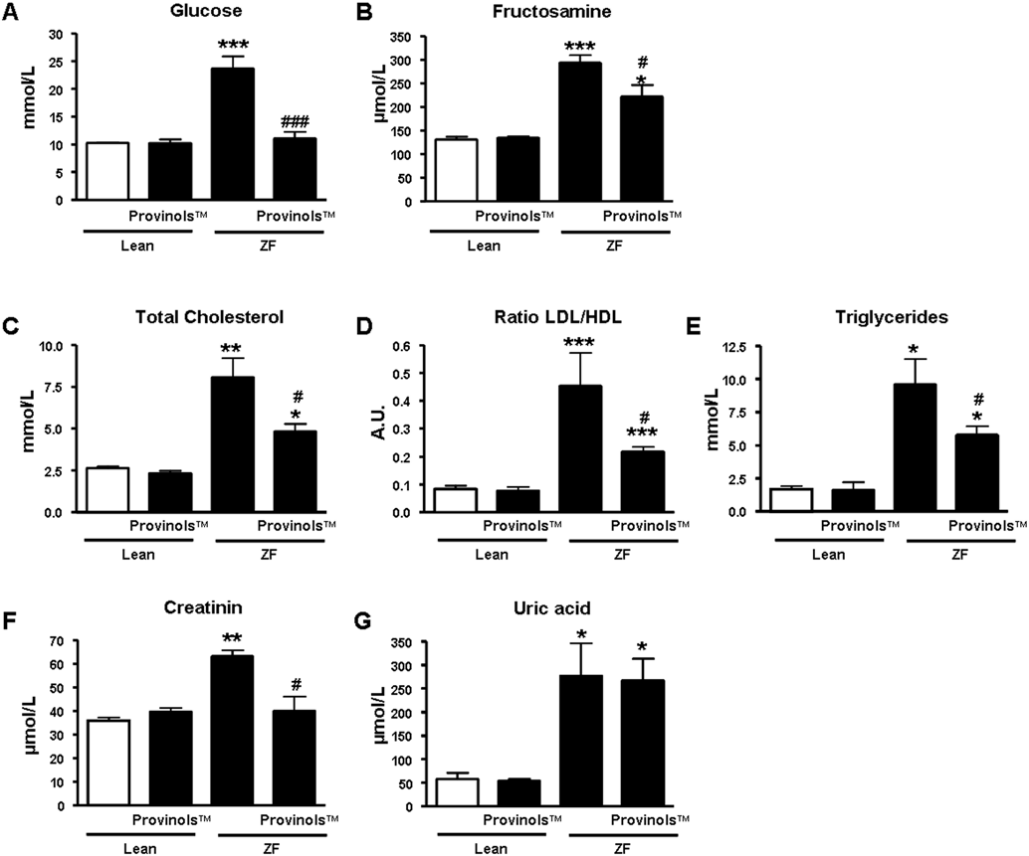
Provinols™ improve metabolic parameters in ZF rats. Circulating levels of glucose (A), fructosamine (B), total cholesterol (C), ratio between LDL- and HDL-cholesterol (D), triglygerides (E), creatinin (F) and uric acid (G) were evaluated in fasting plasma of rats. Values are means±SEM (*n* = 11–12). *P<0.05, **P<0.01, ***P<0.001 Zucker fatty (ZF) rats *vs.* lean rats; #*P*<0.05, ###*P*<0.001 ZF+Provinols™ rats *vs.* ZF rats.

As expected, ZF rats exhibited high glucose and fructosamine levels compared to their lean littermates (Figure 1A, 1B). Interestingly, Provinols™ normalized both glycemia and fructosamine levels in ZF rats, suggesting an improvement in whole-body glucose homeostasis [24]. In addition, ZF rats displayed a high circulating level of total cholesterol (Figure 1C) as well as an elevated ratio between LDL- and HDL-cholesterol (Figure 1D) compared to lean rats. Interestingly, these two parameters were significantly improved by the Provinols™ treatment in ZF rats. Moreover, ZF rats exhibited high levels of circulating triglycerides compared to lean littermates (Figure 1E), and Provinols™ treatment significantly reduced triglyceride level in these animals. Finally, we observed that ZF rats exhibited increased levels of creatinin (Figure 1F) and uric acid (Figure 1G) compared to their lean controls, and while Provinols™ did bit affect the uric acid levels, it reduced the creatinin levels to those measured in lean controls (Figure 1F).

### Provinols™ improve cardiac parameters in ZF rats

**Figure 2:**
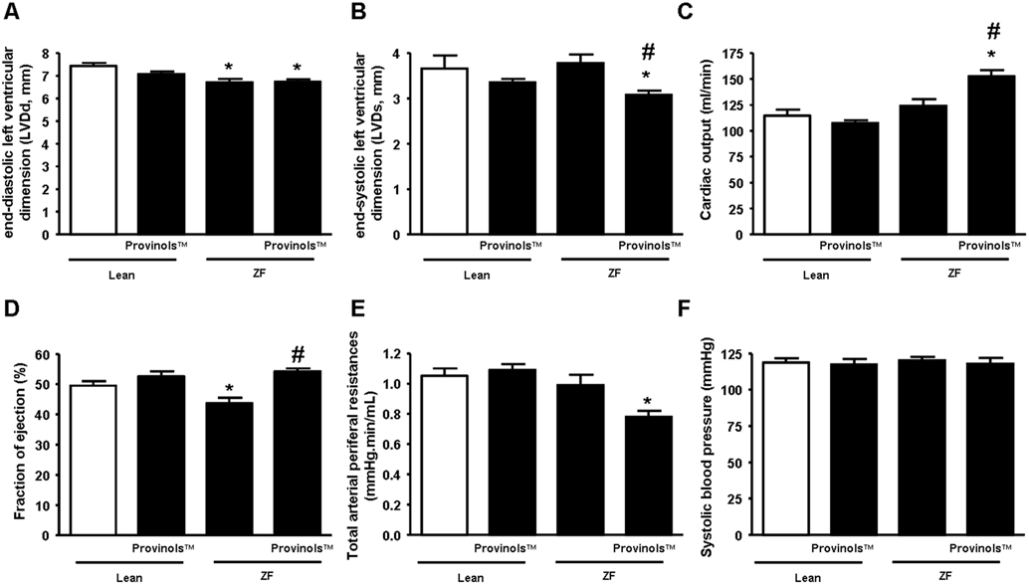
Provinols™ improve cardiac function in ZF rats. Echocardiography measurements of diastolic left ventricular dimension (LVDd), systolic left ventricular dimension (LVDs), cardiac output and fraction of ejection (A–D). Systolic blood pressure was evaluated using tail-cuff technique (E). Total arterial peripheral resistances were calculated from blood pressure and cardiac output (F). Values are means±SEM (*n* = 6). *P<0.05 Zucker fatty (ZF) rats *vs.* lean rats; #*P*<0.05 ZF+Provinols™ rats *vs.* ZF rats.

We investigated the structure and function of the left ventricle of ZF compared to their lean littermates by echocardiography and assessed the effect of Provinols™ (Figure 2). We found that ZF rats displayed a reduced diastolic left ventricular dimension (LVDd) (Figure 2A) compared to lean controls, even though the systolic left ventricular dimension (LVDs) remained the same (Figure 2B). Interestingly, Provinols™ reduced LVDs without affecting LVDd in ZF rats compared to the lean littermates (Figure 2A and 2B), and thus, increased left ventricular fractional shortening.

Moreover, we observed that although the ZF rats presented no difference in cardiac output compared to lean rats (Figure 2C); they exhibited a decreased fraction of ejection (Figure 2D). Importantly, Provinols™ enhanced cardiac output in ZF rats (Figure 2C) and increased the fraction of ejection (Figure 2D) in comparison to their controls.

In addition, we found that Provinols™ significantly decreased arterial peripheral resistance in ZF rats (Figure 2E), while the systolic blood pressure remained the same in both groups of rats (Figure 2F).

### Provinols™ improve endothelial function in both aortas and small mesenteric arteries (SMA) from ZF rats

Endothelial dysfunction, which is a key early factor in the development of atherosclerosis and a predictor of cardiovascular events, has been found in patients with obesity and metabolic syndrome [5], [25]. Thus, we evaluated the endothelial function in both conductance (aorta) and resistance (SMA) arteries from ZF rats and evaluated the potential protective effect of Provinols™.

**Figure 3:**
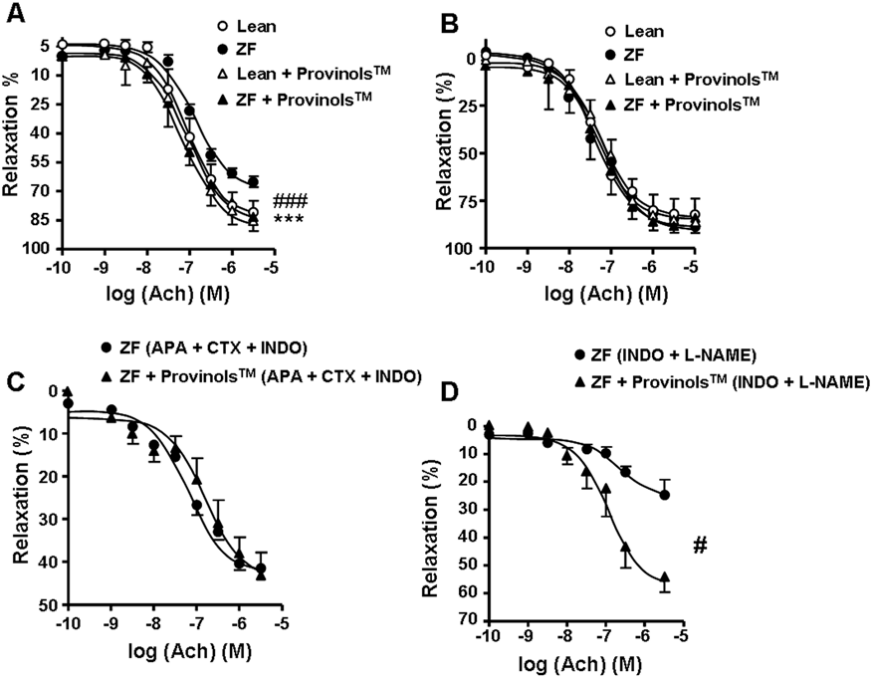
Provinols™ improve endothelial function in ZF rats. Acetylcholine (Ach)-induced relaxation in rat aorta (A), small mesenteric arteries (SMA) (B), SMA in the presence of inhibitors of EDHF and COX components (C) (apamine, APA; charybdotoxin, CTX; indomethacin, INDO), SMA in the presence of inhibitors of NO and COX components (D) (INDO, L-NAME). Results are expressed as a percentage of relaxation. Values are means±SEM (*n* = 6). *P<0.05, ***P<0.001 Zucker fatty (ZF) rats *vs.* lean rats; #*P*<0.05, ###*P*<0.001 ZF+Provinols™ rats *vs.* ZF rats.

As shown in Figure 3A, aortic rings from ZF rats had reduced endothelium-dependent vasodilator responses to Ach compared to aortic rings from lean controls. Interestingly, Provinols™ significantly corrected this impairment in endothelium-dependent relaxation in aortas from ZF rats (Figure 3A).

In SMA, endothelium-dependent relaxation was the same in all four groups (Figure 3B). To get further insight in the component that may be affected upon Provinols™ treatment, we analyzed the effect of pharmacological inhibitors in SMA from ZF rats only. Interestingly, while Provinols™ did not affect the NO-component of the response to Ach agonist (Figure 3C), it markedly enhanced its EDHF-component in SMA taken from ZF rats (Figure 3D) compared to non-treated ZF rats.

Altogether, these findings indicate that Provinols™ improve endothelial function in aortas from ZF rats and in SMA in response to pharmacological stimuli.

### Provinols™ increase arterial NO release in ZF rats by activating eNOS

Given the important role of NO in the mediation of endothelium-dependent relaxation in vessels, we evaluated here the molecular pathways implicated to its production upon Provinols™ treatment in different arteries.

**Figure 4:**
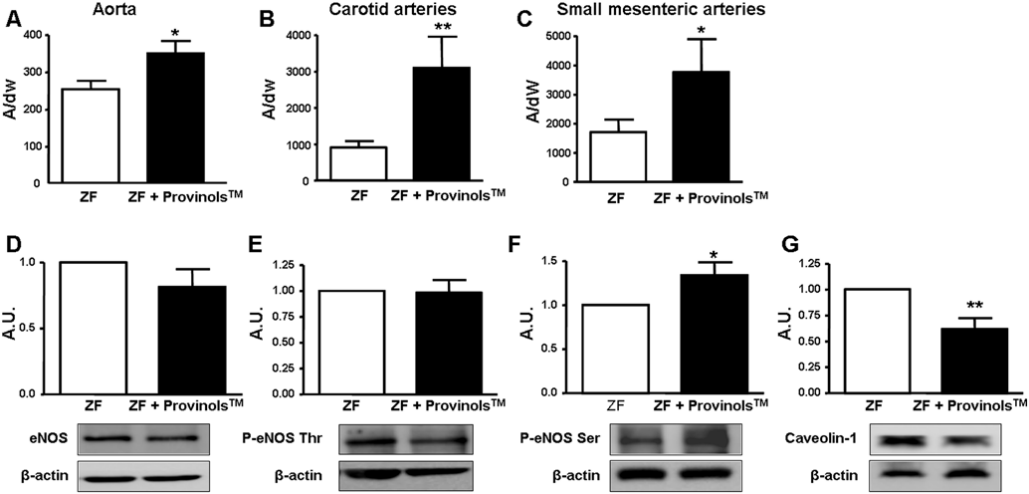
Provinols™ increase NO vascular release in vessels from ZF rats. NO production in aorta (A), carotid arteries (B) and small mesenteric arteries (C) from Zucker fatty (ZF) and ZF+Provinols™ rats. Values are expressed as units of amplitude (A)/mg weight of dried tissue (dw). Western blots revealing expression of eNOS (D), phosphorylation of eNOS at Thr495 (E), and NOS Ser1177 (F) and expression of caveolin-1 (G) in aorta expressed as arbitrary units (A.U.). (*n* = 6). *P<0.05, **P<0.01 *vs.* ZF rats.

Treatment of ZF rats with Provinols™ increased NO production in aorta (Figure 4A), carotid artery (Figure 4B) and SMA (Figure 4C), suggesting that the improvement in endothelial function observed in these arteries is associated with a higher bio-availability of NO. In order to assess the mechanism(s) of NO release, we studied the NO signaling pathway in rat aorta. Although the Provinols™ treatment did not modify either expression (Figure 4D) nor phosphorylation of eNOS at the inhibitor site (Thr 495, Figure 4E), it significantly enhanced its phosphorylation at the activator site (Ser 1177, Figure 4F), suggesting that Provinols™ enhanced eNOS activity. Importantly, Provinols™ reduced expression of caveolin-1 (Figure 4G), a protein known to inactivate eNOS by cell membrane sequestration.

### Provinols™ decrease arterial O_2_^−^ release in ZF rats by decreasing the expression of the Nox-1 subunit of NADPH oxidase

Oxidative stress, which is one of the causative factors of endothelial dysfunction, is thought to play a major role in the occurrence and complications of obesity and metabolic syndrome [26]. Hence, we evaluated the vascular production of O_2_^−^ in ZF rats and its modulation by Provinols™ treatment.

**Figure 5:**
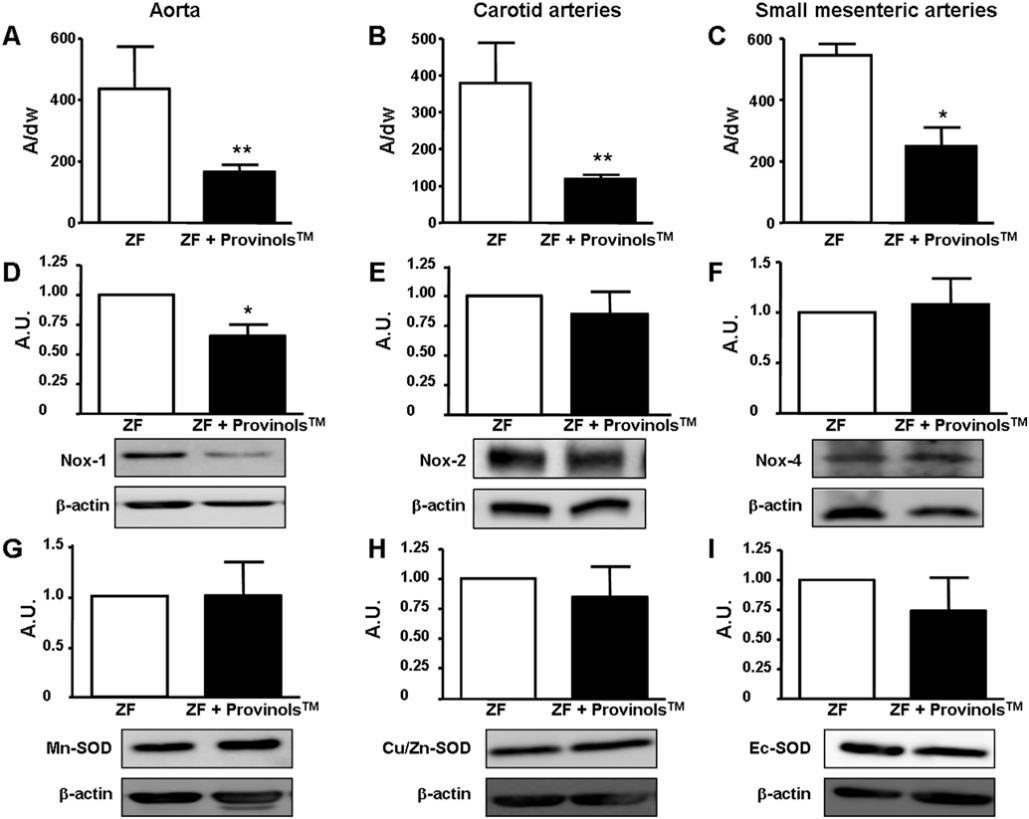
Provinols™ reduce O_2_^−^ vascular release in vessels from ZF rats. O_2_^−^ production in aorta (A), carotid arteries (B) and small mesenteric arteries (C) from Zucker fatty (ZF) and ZF+Provinols™ rats. Values are expressed as units of amplitude (A)/mg weight of dried tissue (dw). Western blots revealing expression of Nox-1 (D), Nox-2 (E), Nox-4 (F), Mn-SOD (G), Cu/Zn-SOD (H) and Ec-SOD (I) in aorta expressed as arbitrary units (A.U.). (*n* = 6). *P<0.05, **P<0.01 *vs.* ZF rats.

EPR study demonstrated that Provinols™ significantly reduced O_2_^−^ production in aorta (Figure 5A), carotid arteries (Figure 5B) and SMA (Figure 5C) from ZF rats, indicating a decrease in oxidative stress favorable to an increase in NO bio-availability.

We evaluated the expression of membrane subunits of NADPH oxidase (Nox-1, Nox-2 and Nox-4), a major source of cellular O_2_^−^, in aortas from ZF rats. Provinols™ treatment reduced the expression of Nox-1 subunit (Figure 5D) without affecting that of Nox-2 or Nox-4 (Figure 5E, 5F). To evaluate the effect of Provinols™ on the vascular capacity to dismutate O_2_^−^, we examined expression of different isoforms of superoxide dismutase (SOD). Provinols™ did not significantly modify Mn-SOD, Cu/Zn-SOD or Ec-SOD expressions (Figure 5G–5I).

## Discussion

In the present study, we demonstrate that Provinols™ leads to an improvement of glucose and lipid metabolism in ZF rats, enhances cardiac function and decreases peripheral resistance. In addition, Provinols™ improves endothelium-dependent relaxation in both aorta and SMA from ZF rats and this is accompanied with an increase of vascular NO production and with a decrease of O_2_^−^ release, resulting in an increase of NO bioavailability. These effects are associated with an increase in eNOS activity, and a decrease in caveolin-1 expression, in addition to a decreased expression of Nox-1 NADPH oxidase subunit. Interestingly, these results show that Provinols™ leads to an overall improvement of obesity-associated alterations, including glucose and lipid metabolism, as well as endothelial and cardiac functions, by mechanisms not related to those linked to a decrease of body weight gain in ZF rats.

Here, we provide evidence that Provinols™ treatment was effective in acting on the parameters linked to insulin resistance (glucose and fructosamine levels) in ZF rats compared to their lean littermates. These effects most likely reduced the deleterious consequences of increased glycaemia, including the formation of advanced glycosylated end products that not only increase oxidative stress but also induce endothelial dysfunction. In accordance with the present study, other compounds, such as resveratrol, present in red wine exhibit hypoglycaemic effects in streptozotocin-induced diabetic rats [27] and in obese ZF rats [28]. Moreover, Napoli et al. [29] showed that red wine consumption improves insulin resistance in type 2 diabetic patients.

With regard to lipid lowering properties of Provinols™, the present study shows that Provinols™ reduced circulating triglycerides and total cholesterol levels, as well as the ratio between LDL- and HDL-cholesterol in ZF rats compared to lean controls. Some animal studies suggest that polyphenols may reduce cholesterol absorption due to the interaction of these compounds with cholesterol carriers and transporters present across the brush border membrane [30], [31]. Very recently, Rivera et al. [28] have shown that long-term resveratrol administration reduces plasma cholesterol and triglyceride levels in ZF rats. Also, Vinson et al. [32] reported that red wine polyphenol compounds or grape juices reduce the plasma concentration of lipids in hamsters. This is consistent with the study in humans, in which short-term ingestion of purple grape juice has been shown to reduce LDL susceptibility to oxidation in patients with coronary artery disease [33]. Indeed, consumption of red wine also reduces LDL oxidation leading to limited plaque formation in humans [34]. LDL oxidation was not assessed here but we clearly demonstrated that Provinols™ improves the balance between LDL- and HDL-cholesterol in obese rats.

In regards to the structure and function of the left ventricle of ZF rats, we showed that Provinols™ reduced the LVDs without affecting the LVDd and thus improved fractional shortening. Lee and coworkers [35] suggested that ZF rats exhibited reduced LVDs and LVDd compared to their lean littermates; however, the fractional shortening was not different amongst the two groups. The improvement in LVDs and fractional shortening induced by Provinols™ was also accompanied with an increase in fraction of ejection and cardiac output, illustrating an improvement in systolic function. In addition, Provinols™ reduced total arterial peripheral resistance leading to the absence of modification in blood pressure. Altogether, these findings highlight the beneficial effects of Provinols™ on cardiac function inasmuch cardiomyopathic changes are noted in obesity although it cannot be isolated and attributed to any specific factor, such as volume overload, oxidative stress, blood pressure changes, or other unclear factors.

Endothelial dysfunction and hypertension are closely related to obesity and/or insulin resistance [36]. Although many studies reported that the endothelium-dependent relaxation in response to Ach is paradoxically preserved or even enhanced in ZF rats relative to their lean littermates [37], impaired Ach-induced vasodilatation has also been described [38]. Numerous investigations have indicated that red wines, grape juices, red wine polyphenolic and grape skin extracts are potent endothelium-dependent vasodilators [9], [10], [39]. Herein, we demonstrated that the Ach-induced relaxation was impaired in aortas from ZF rats compared to lean controls, and importantly was completely corrected by Provinols™ treatment. This improvement in endothelial function was attributable to the increase in NO bio-availability as given by the concomitant increase in NO and decrease in O_2_^−^ releases in vessels taken from Provinols™-treated ZF rats compared to non-treated ZF rats. In accordance with our previous study [40], Provinols™ treatment enhanced activity of eNOS and decreased the expression of caveolin-1, a protein known to sequestrate eNOS. In addition, we showed here that Provinols™ decreases NADPH oxidase Nox-1 subunit expression, leading to a decrease in O_2_^−^ release from aorta. In agreement with our data, Sarr et al. [41] reported that red wine polyphenols prevented the angiotensin II-induced expression of Nox-1 subunit in rat aorta.

Of note is that increased EDHF component was highlighted in response to Ach-induced vasodilatation (chemical stimulation), although no difference among the groups has been observed in SMA. Blockade of COX and EDHF components using INDO and charybdotoxin plus apamin, reduced the response to Ach in both groups of ZF rats to the same extent, even though the bio-availability of NO was increased in vessels from Provinols™-treated animals, suggesting that the amount of NO released in non-treated ZF rats is sufficient to induce the maximal effect. Interestingly, when the NO and COX components were inhibited using INDO and L-NAME, the endothelium-dependent relaxation in response to Ach was decreased in ZF versus Provinols™-treated ZF rats suggesting that Provinols™ treatment increases EDHF-mediated relaxation in SMA. Previous studies have shown that red wine polyphenols induced EDHF-dependent relaxation in porcine coronary arteries [39], [42]. These results underlined an increased EDHF contribution on Ach response upon Provinols™ after blockade of both NO and COX pathways as a possible compensatory mechanism in the same way to those shown in our previous studies in human resistance arteries [43].

The treatment of metabolic diseases and obesity-related complications are currently based on the treatment of their individual components, e.g. hypertension and insulin resistance. Due to the multi-factorial properties of Provinols™, they may be a good candidate for prevention and treatment of metabolic syndrome and reduction of cardiovascular risk. Our present study provides strong evidence for beneficial effects of red wine polyphenols on both, vascular and cardiac functions in ZF rats, suggesting that they may therefore be of therapeutic benefit in the future and may represent a new class of medicinal products against obesity-associated diseases.
